# Emergence of *Streptococcus pyogenes emm*102 Causing Toxic Shock Syndrome in Southern Taiwan during 2005–2012

**DOI:** 10.1371/journal.pone.0081700

**Published:** 2013-12-09

**Authors:** Jiun-Nong Lin, Lin-Li Chang, Chung-Hsu Lai, Hsi-Hsun Lin, Yen-Hsu Chen

**Affiliations:** 1 Graduate Institute of Medicine, College of Medicine, Kaohsiung Medical University, Kaohsiung, Taiwan; 2 Department of Critical Care Medicine, E-Da Hospital, I-Shou University, Kaohsiung, Taiwan; 3 Division of Infectious Diseases, Department of Internal Medicine, E-Da Hospital, I-Shou University, Kaohsiung, Taiwan; 4 Department of Microbiology, Faculty of Medicine, College of Medicine, Kaohsiung Medical University, Kaohsiung, Taiwan; 5 Institute of Clinical Medicine, National Yang-Ming University, Taipei, Taiwan; 6 School of Medicine, College of Medicine, Kaohsiung Medical University, Kaohsiung, Taiwan; 7 Division of Infectious Diseases, Department of Internal Medicine, Kaohsiung Medical University Hospital, Kaohsiung, Taiwan; Rockefeller University, United States of America

## Abstract

**Background:**

Streptococcal toxic shock syndrome (STSS) is an uncommon but life-threatening disease caused by *Streptococcus pyogenes*.

**Methods:**

To understand the clinical and molecular characteristics of STSS, we analyzed clinical data and explored the *emm* types, superantigen genes, and pulsed-field gel electrophoresis of causative *S. pyogenes* isolates obtained between 2005 and 2012.

**Results:**

In total, 53 patients with STSS were included in this study. The median age of the patients was 57 years (range: 9–83 years), and 81.1% were male. The most prevalent underlying disease was diabetes mellitus (45.3%). Skin and soft-tissue infection accounted for 86.8% of STSS. The overall mortality rate was 32.1%. Underlying diseases had no statistical impact on mortality. A total of 19 different *emm* types were identified. The most prevalent *emm* type was *emm*102 (18.9%), followed by *emm*11 (17%), *emm*1 (11.3%), *emm*87 (9.4%), and *emm*89 (7.5%). There was no statistically significant association between *emm* type and a fatal outcome. Among the superantigen genes, *speB* was the most frequently detected one (92.5%), followed by *smeZ* (90.6%), *speG* (81.1%), *speC* (39.6%), and *speF* (39.6%). The majority of *emm*102 strains were found to have *speB*, *speC*, *speG*, and *smeZ*. The presence of *speG* was negatively associated with a fatal outcome (*P* = 0.045).

**Conclusions:**

Our surveillance revealed the emergence of uncommon *emm* types, particularly *emm*102, causing STSS in southern Taiwan. Characterization of clinical, epidemiological, and molecular characteristics of STSS will improve our understanding of this life-threatening disease.

## Introduction

Streptococcal toxic shock syndrome (STSS) is a rare but potentially fatal disease that is characterized by the sudden onset of shock and multiple organ failure [1]. It is caused by toxin-producing strains of *Streptococcus pyogenes* (group A streptococcus [GAS]) [2]. This microorganism exists ubiquitously in the environment and can cause a wide variety of human infections that range from mild pharyngitis and cellulitis to life-threatening diseases, such as necrotizing fasciitis, puerperal sepsis, pneumonia, bacteremia, and STSS [3]. Although a significant reduction in the incidence of invasive GAS infection occurred in the early 20th century, a resurgence of this severe infectious disease has been noted since the late 1980s [4], [5].

Several virulence determinants have been found to be involved in the pathogenesis of invasive GAS infections. Among these virulence factors, superantigens (SAgs) are known to play a pivotal role in the pathogenesis of STSS [2], [6], [7]. SAgs can bypass the conventional process of antigen presentation and interact with antigen-presenting cells and T cells simultaneously. This reaction results in T cell proliferation and then produces massive amounts of cytokines that lead to rash, fever, capillary leakage, organ failure, and subsequent shock [2], [6], [7]. Currently, at least 11 distinct SAgs have been identified in GAS, including SpeA, SpeC, SpeG, SpeH, SpeI, SpeJ, SpeK, SpeL, SpeM, SSA, and SmeZ [8], [9]. The proteins SpeB and SpeF were initially described as SAgs, but they were found to have the properties of cysteine protease and DNase, respectively [10], [11]. Some studies contended that their mitogenic activity was due to contamination with small amounts of the exceptionally potent SmeZ [10], [11]. Allelic variations were found in SAg genes, including *speA*, *speC*, *speG*, *ssa*, and *smeZ* [12]–[15]. For example, 6 allelic variants of the *speA* have been described (*speA1*-*speA6*) [13]. *smeZ* displays more extensive allelic variation and at least 50 different *smeZ* alleles have been deposited in GenBank [14], [15]. The varied alleles may exhibit different antigenic properties and superantigenic activity [13], [14].

M protein, which is encoded by the *emm* gene, is also a major virulence factor of GAS. Traditionally, GAS strains are classified based on M protein serotypes. In recent years, *emm* gene sequencing has widely replaced M protein serotyping as the gold standard for the epidemiological surveillance of GAS infections [16], [17]. To date, more than 250 different *emm* types have been identified [18]. The global distribution of GAS *emm* types varies across different geographic regions. For example, *emm*1, *emm*12, and *emm*28 were the leading types isolated from high-income countries; *emm*12, *emm*st62, and *emm*75 were the top 3 strains in Africa; and *emm*55, nontypable strains, and *emm*11 ranked first in the Pacific region [5]. In Taiwan, the epidemiology of GAS infections is quite different from other countries [19]–[25]. Recent studies in Taiwan have shown an emergence of uncommon *emm* types causing invasive GAS infections, including *emm*11, *emm*102, and *emm*106 [26]–[28]. These *emm* data are important for the development of a GAS vaccine, as some experimental vaccines are based on M proteins of GAS [29]–[31].

Studies describing STSS in Taiwan are limited, and most are case reports or case series. To provide better insight into the current epidemiological information and molecular characteristics of STSS in Taiwan, we analyzed clinical data and explored the *emm* types, SAg genes, and pulsed-field gel electrophoresis (PFGE) of causative GAS isolates obtained between 2005 and 2012. Our study revealed a particular epidemiology and the molecular characteristics of STSS in southern Taiwan.

## Materials and Methods

### Ethics Statement

This study was approved by the Institutional Review Board of E-Da Hospital. The need for patient's informed consent was waived by the Institutional Review Board of E-Da Hospital as the retrospective analysis of anonymous data posed no more than minimal risk of harm to subjects and involved no procedures for which written consent was normally required outside of the research context.

### Study Design

This study was conducted at E-Da Hospital, which is a 1,100-bed university-affiliated hospital that serves more than 2 million people in southern Taiwan. The computer database was searched to identify patients with positive GAS cultures between January 2005 and December 2012. The medical charts with positive GAS cultures were reviewed, and those patients who met the diagnostic criteria for STSS were included in this study. Demographic information and clinical characteristics were collected from the medical records. *emm* typing, SAg gene identification, and PFGE were performed to investigate the epidemiological and molecular characteristics of the causative microorganisms.

### Case Definition

STSS was diagnosed according to the following criteria [1]: isolation of GAS from a clinically striking specimen in patients with hypotension (systolic blood pressure <90 mm Hg) and multiple organ failure (≥2 organs), including coagulopathy, liver dysfunction, acute respiratory distress syndrome, a generalized erythematous macular rash, and soft-tissue necrosis.

### Identification and Culture of GAS

All GAS isolates were identified by the clinical laboratory of E-Da Hospital using standard methods [32]. The isolates were then stored in −80°C glycerol. The criteria for GAS identification included β-hemolysis on sheep blood agar, a positive Gram stain, a negative catalase test, a positive pyrrolidonyl arylamidase test, and agglutination with Lancefield group A antiserum. For molecular experiments in our study, the stored isolates were thawed and then subcultured at 37°C in Todd-Hewitt broth (Difco) supplemented with 0.5% yeast extract (THY). *Streptococcus pyogenes* ATCC 12344 was used as a control strain.

### *emm* Typing

The *emm* typing of GAS was performed according to the protocol from the Centers for Disease Control and Prevention (CDC) [33]. The primers for polymerase chain reaction (PCR) of *emm* genes are listed in Table 1. PCR was performed using the GeneAmp PCR System 9700 (Applied Biosystems, Foster City, CA, USA), and the amplicons were then sent to a biotechnology company (Genomics BioSci & Tech Corp., Taipei, Taiwan) for DNA sequencing using the Applied Biosystems 3730xl DNA Analyzer. The 5′ *emm* sequence data were submitted to the *emm* database on the CDC website to determine the *emm* type of each isolate [34].

**Table 1:** List of Primers for Polymerase Chain Reaction Used in This Study.

Gene	Sequence (5′→3′)		Reference
	Forward primer	Reverse primer	
*emm*	TATT(C/G)GCTTAGAAAATTAA	GCAAGTTCTTCAGCTTGTTT	[33]
*speA*	TAAGAACCAAGAGATGG	ATTCTTGAGCAGTTACC	[35]
*speB*	AAGAAGCAAAAGATAGC	TGGTAGAAGTTACGTCC	[35]
*speC*	GATTTCTACTTATTTCACC	AAATATCTGATCTAGTCCC	[35]
*speF*	TACTTGGATCAAGACG	GTAATTAATGGTGTAGCC	[35]
*speG*	AGAAACTTATTTGCCC	TAGTAGCAAGGAAAAGG	[35]
*speH*	AGATTGGATATCACAGG	CTATTCTCTCGTTATTGG	[35]
*speI*	CTTTGGAGTATTCTCCTCCC	CTCTCTCTGTCACCATGTCC	[8]
*speJ*	ATCTTTCATGGGTACG	TTTCATGTTTATTGCC	[35]
*speK*	GTCATATCATGTTGTATGCAA	GTTTAAGTGAACATCAAAGTG	[8]
*speL*	TTAGGATGGTTTCTGCGGAAGAGAC	TTCCTCTTTCTCGCCTGAGCCGTG	[8]
*speM*	GCTCTATACACTACTGAGAGTGTC	CATATCAATCGTTTCATTATCTG	[8]
*ssa*	GTGTAGAATTGAGGTAATTG	TAATATAGCCTGTCTCGTAC	[35]
*smeZ*	GAAGTAGATAATAATTCCCTTCTAAGG	AGTCAATTTCTATATCTAAATGCCC	[8]

### SAg Genotyping

All isolates were examined for the presence of SAg genes, including *speA*, *speB*, *speC*, *speF*, *speG*, *speH*, *speI*, *speJ*, *speK*, *speL*, *speM*, *ssa*, and *smeZ*. The PCR primers for gene profiling are listed in Table 1. The PCR process was modified from the protocol described previously [8], [19], [35]. As some SAg genes have only approximate product sizes, it is difficult to distinguish these amplicons from those with similar sizes in the same multiplex PCR tube. Therefore, *speA*, *speB*, *speG*, *speH*, *speJ*, and *ssa* were detected using multiplex PCR and *speC*, *speF*, *speI*, *speK*, *speL*, *speM*, and *smeZ* were identified using single PCR.

### PFGE Analysis

PFGE was performed according to a protocol described previously [36]. All isolates were digested with *Sma*I, and the isoschizomer Cfr9I was used for the isolates that did not digest with *Sma*I [37]. The digested fragments of DNA were separated by CHEF Mapper XA Chiller System (Bio-Rad, Hercules, CA, USA). The generated fragments were analyzed using the Unweighted Pair Group Method with the arithmetic mean algorithm by GelCompar II software version 6 (Applied Maths, Austin, TX, USA) to evaluate their similarity and epidemiological relationships. PFGE clusters were defined as isolates sharing at least 80% similarity [37].

### Data Analysis

The data were analyzed using SPSS 14.0 (SPSS Inc., Chicago, IL, USA). In the univariate analysis, continuous and categorical data were analyzed using Student's *t*-test and the Mantel-Haenszel test, respectively. The odds ratio (OR), 95% confidence interval (CI), and *P* value were calculated for each variable. To identify the risk factors and control for potential confounders, underlying conditions that could contribute to STSS and were associated with a level of significance of ≤0.25 in univariate analyses were included in a logistic regression model for multivariate analysis using backward stepwise methods by likelihood ratio. Differences were considered statistically significant if the two-tailed *P* value was <0.05.

## Results

### Demographic Data

**Figure 1:**
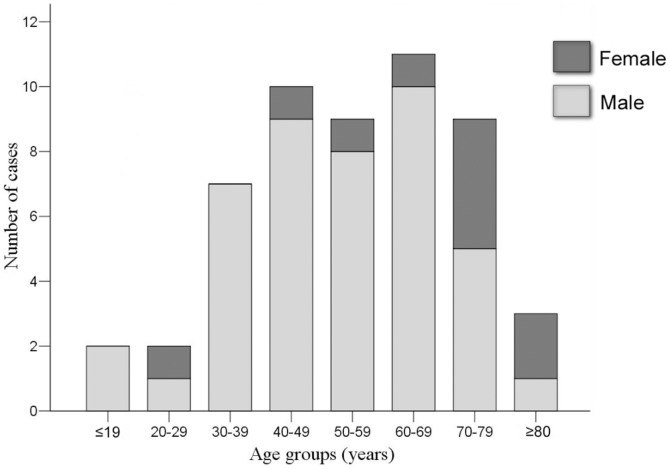
The distribution of age and sex in patients with streptococcal toxic shock syndrome. Males accounted for 81.1% of patients. Most patients (73.6%) were between 40 and 79 years old.

During the study period, 53 patients were diagnosed with STSS (Table 2). All patients presented to the emergency department (ED), and the indicated incidence was 10.4 cases per 100,000 ED visits. Among the 53 patients, 43 (81.1%) were male and 10 (18.9%) were female. The median age of the included patients was 57 years (range: 9–83 years), with a mean ± standard deviation (SD) of 54.7±17.4 years. Most patients (73.6%) were between 40 and 79 years old (Figure 1).

**Table 2:** Demographic Characteristics, Underlying Diseases, Clinical Conditions, and Sources of Infection among 53 Cases of Streptococcal Toxic Shock Syndrome.

Characteristic	No. (%)
Age (mean ±SD) (yr)	54.7±17.4
Sex	
Male	43 (81.1)
Female	10 (18.9)
Underlying disease	
Diabetes mellitus	24 (45.3)
Hypertension	20 (37.7)
Congestive heart failure	5 (9.4)
Malignancy	2 (3.8)
Liver cirrhosis	12 (22.6)
Gout	17 (32.1)
HIV^a^ infection	1 (1.9)
Source of infection	
Skin and soft-tissue infection	46 (86.8)
Peritonitis	1 (1.9)
Osteomyelitis	1 (1.9)
Primary bacteremia	5 (9.4)
Laboratory	
White blood cell count (cells/mm^3^)	17,199±10,647
Hemoglobin (g/dl)	12.1±2.2
Platelet count (×1,000 cells/mm^3^)	161±102
Serum creatinine (mg/dl)	2.6±1.4
Intensive care unit admission	37 (69.8)
Mortality	17 (32.1)

a HIV, human immunodeficiency virus.

### Clinical Characteristics

The majority (86.8%) of patients had chronic diseases (Table 2). The most prevalent underlying disease was diabetes mellitus (45.3%), followed by hypertension (37.7%), gout (32.1%), and liver cirrhosis (22.6%). The most common infection that complicated STSS was skin and soft-tissue infection (86.8%), followed by primary bacteremia (bacteremia without an identified focus; 9.4%). Among those patients with skin and soft-tissue infection, 80.4% received surgical interventions. The mean ±SD for white blood cell (WBC) counts, hemoglobin, platelet counts, and serum creatinine are shown in Table 2.

### *emm* Type

**Figure 2:**
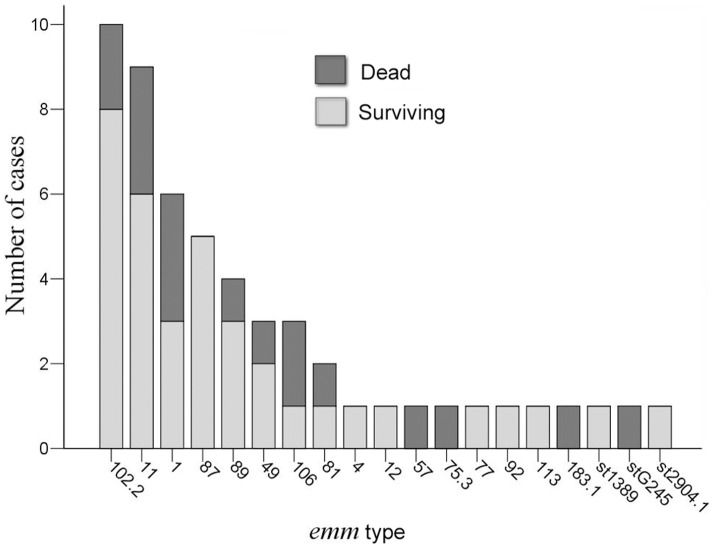
The distribution of *emm* types in *Streptococcus pyogenes* and fatality in patients with streptococcal toxic shock syndrome. A total of 19 different *emm* types were identified. The most prevalent *emm* type was *emm*102 (18.9%), followed by *emm*11 (17%), *emm*1 (11.3%), *emm*87 (9.4%), and *emm*89 (7.5%). Patients infected with GAS *emm*1 (50%) and *emm*106 (66.7%) had a higher mortality rate, while patients infected with *emm*102 had a lower fatality rate of 20%.

The *emm* types of GAS that were identified as causing STSS are shown in Figure 2. A total of 19 different *emm* types were identified. The most prevalent *emm* type was *emm*102 (18.9%), followed by *emm*11 (17%), *emm*1 (11.3%), *emm*87 (9.4%), and *emm*89 (7.5%). These 5 *emm* types accounted for 64.2% of all isolates.

### Outcome

Approximately 70% of patients were admitted to intensive care units. All the patients infected with *emm*106 and 66.7% of patients infected with *emm*1 were admitted to intensive care units. However, only half of patients infected with *emm*102 needed to be admitted to intensive care units. There was no significant association between *emm* types and intensive care unit admission (*emm*102 vs. *emm*1, *P* = 0.633; *emm*102 vs. *emm*106, *P* = 0.231). The overall mortality rate for patients with STSS was 32.1%. Underlying diseases had no statistical impact on mortality, either in the univariate or multivariate analysis (Table 3). Patients presenting with WBC≥10,000 cells/mm^3^ were predisposed to have a lower mortality rate (OR = 0.27 [95% CI, 0.08 to 0.96]; *P* = 0.043). In contrast, patients with a lower platelet count (<100,000 cells/mm^3^) were significantly associated with a fatal outcome (OR = 3.68 [95% CI, 1.04 to 13]; *P* = 0.043). Patients infected with GAS *emm*1 and *emm*106 had a higher mortality rate (50% and 66.7%, respectively), while patients infected with *emm*102 had a lower fatality rate of 20%. However, there was no significant association between the *emm* type and a fatal outcome (Table 4).

**Table 3:** Clinical Factors for Mortality in Patients with Streptococcal Toxic Shock Syndrome.

Factor	Mortality		Univariate analysis	
	With factor	Without factor	OR^a^ (95% CI^b^)	*P* value
Age ≥65 yrs	6 (30)	11 (33.3)	0.86 (0.26–2.84)	0.801
Sex, male	13 (30.2)	4 (40)	0.65 (0.16–2.7)	0.553
Underlying disease				
Diabetes mellitus^c^	5 (20.8)	12 (41.4)	0.37 (0.11–1.28)	0.116
Hypertension^d^	4 (20)	13 (39.4)	0.39 (0.11–1.41)	0.149
Congestive heart failure	1 (20)	16 (33.3)	0.5 (0.05–4.85)	0.55
Malignancy	1 (50)	16 (31.4)	2.19 (0.13–37.22)	0.588
Liver cirrhosis	5 (41.7)	12 (29.3)	1.73 (0.46–6.53)	0.421
Gout	4 (23.5)	13 (36.1)	0.54 (0.15–2.02)	0.363
HIV^e^ infection	1 (100)	16 (30.8)	-	0.999
Laboratory test				
White blood cell count ≥10,000 cells/mm^3^	9 (23.7)	8 (53.3)	0.27 (0.08–0.96)	0.043
Hemoglobin <10 g/dl	5 (45.5)	12 (28.6)	2.08 (0.53–8.14)	0.291
Platelet count <100,000 cells/mm^3^	8 (53.3)	9 (23.7)	3.68 (1.04–13)	0.043
Serum creatinine ≥2.5 mg/dl	7 (35)	10 (30.3)	1.24 (0.38–4.04)	0.723

a OR, odds ratio.

b CI, confidence interval.

c,d Multivariate logistic regression analysis using backward stepwise methods by likelihood ratio: diabetes mellitus, OR = 0.37 (95% CI, 0.11 to 1.28); *P* = 0.116; hypertension, OR = 0.46 (95% CI, 0.12–1.73); *P* = 0.248.

e HIV, human immunodeficiency virus.

**Table 4:** Association between *emm* Types and Superantigen Genes of *Streptococcus pyogenes* and Mortality in Toxic Shock Syndrome.

Factor	Mortality		OR^a^ (95% CI^b^)	*P* value
	With factor	Without factor		
*emm* type				
*emm*102	2 (20)	15 (34.9)	0.47 (0.09–2.48)	0.372
* emm*11	3 (33.3)	14 (31.8)	1.07 (0.23–4.92)	0.929
* emm*1	3 (50)	14 (29.8)	2.36 (0.42–13.14)	0.328
* emm*87	0	17 (35.4)	-	-
* emm*89	1 (25)	16 (32.7)	0.69 (0.07–7.14)	0.754
Superantigen gene				
* speA*	3 (50)	14 (29.8)	2.36 (0.42–13.14)	0.328
* speB*	15 (30.6)	2 (50)	0.44 (0.06–3.43)	0.434
* speC*	8 (38.1)	9 (28.1)	1.57 (0.49–5.07)	0.448
* speF*	7 (33.3)	10 (31.3)	1.1 (0.34–3.56)	0.874
* speG*	11 (25.6)	6 (60)	0.23 (0.05–0.97)	0.045
* speH*	3 (27.3)	14 (33.3)	0.75 (0.17–3.28)	0.702
* speI*	5 (55.6)	12 (27.3)	3.33 (0.76–14.54)	0.109
* speJ*	5 (62.5)	12 (26.7)	4.58 (0.95–22.17)	0.058
* speK*	1 (12.5)	16 (35.6)	0.26 (0.03–2.3)	0.225
* speL*	2 (100)	15 (29.4)	-	-
* speM*	2 (50)	15 (30.6)	2.27 (0.29–17.64)	0.434
* ssa*	1 (100)	16 (30.8)	-	-
* smeZ*	15 (31.3)	2 (40)	0.68 (0.1–4.52)	0.691

a OR, odds ratio.

b CI, confidence interval.

### SAg Genes

The SAg genes of different *emm* types are summarized in Table 5. The mean number of SAg genes was 4.36 per isolate. *speB* was the most frequently detected SAg gene (92.5%), followed by *smeZ* (90.6%), *speG* (81.1%), *speC* (39.6%), and *speF* (39.6%). The *speA* gene was carried by 83.3% of *emm*1 and 11.1% of *emm*11, but it was not identified in the other *emm* types. The *smeZ* gene was detected in all *emm* types except *emm*49 and *emm*81. The majority of *emm*102 isolates were found to have *speB*, *speC*, *speG*, and *smeZ*. Most *emm*11 carried *speB*, *speG*, and *smeZ*. The presence of *speG* was negatively associated with a fatal outcome (OR = 0.23 [95% CI, 0.05 to 0.97]; *P* = 0.045) (Table 4).

**Table 5:** Superantigen Genes in *Streptococcus pyogenes* That Caused Toxic Shock Syndrome.

*emm* type	No. (%) of isolates	No. (%) of positive isolates												
		*speA*	*speB*	*speC*	*speF*	*speG*	*speH*	*speI*	*speJ*	*speK*	*speL*	*speM*	*ssa*	*smeZ*
102	10 (18.9)	0	9 (90)	8 (80)	5 (50)	9 (90)	7 (70)	0	2 (20)	0	0	0	0	10 (100)
11	9 (17)	1 (11.1)	9 (100)	2 (22.2)	3 (33.3)	7 (77.8)	0	2 (22.2)	0	0	1 (11.1)	0	0	9 (100)
1	6 (11.3)	5 (83.3)	5 (83.3)	0	3 (50)	5 (83.3)	0	0	2 (33.3)	0	0	0	0	6 (100)
87	5 (9.4)	0	5 (100)	0	3 (60)	5 (100)	0	0	0	5 (100)	0	0	0	5 (100)
89	4 (7.5)	0	4 (100)	4 (100)	0	3 (75)	0	0	2 (50)	0	0	0	0	4 (100)
49	3 (5.7)	0	3 (100)	1 (33.3)	0	2 (66.7)	0	3 (100)	0	0	0	0	0	0
106	3 (5.7)	0	3 (100)	2 (66.7)	1 (33.3)	3 (100)	2 (66.7)	2 (66.7)	0	0	0	0	0	3 (100)
81	2 (3.8)	0	2 (100)	0	1 (50)	2 (100)	0	0	0	2 (100)	0	2 (100)	0	0
Other^a^	11 (20.8)	0	9 (81.8)	4 (36.4)	5 (45.5)	7 (63.6)	2 (18.2)	2 (18.2)	2 (18.2)	1 (9.1)	1 (9.1)	2 (18.2)	1 (9.1)	11 (100)
Total (%)	53 (100)	6 (11.3)	49 (92.5)	21 (39.6)	21 (39.6)	43 (81.1)	11 (20.8)	9 (17)	8 (15.1)	8 (15.1)	2 (3.8)	4 (7.5)	1 (1.9)	48 (90.6)

a Included *emm*4 (*n* = 1), *emm*12 (*n* = 1), *emm*57 (*n* = 1), *emm*75 (*n* = 1), *emm*77 (*n* = 1), *emm*92 (*n* = 1), *emm*113 (*n* = 1), *emm*183 (*n* = 1), *emm*st1389 (*n* = 1), *emm*stG245 (*n* = 1), and *emm*st2904 (*n* = 1).

### Clonal Analysis of PFGE

**Figure 3:**
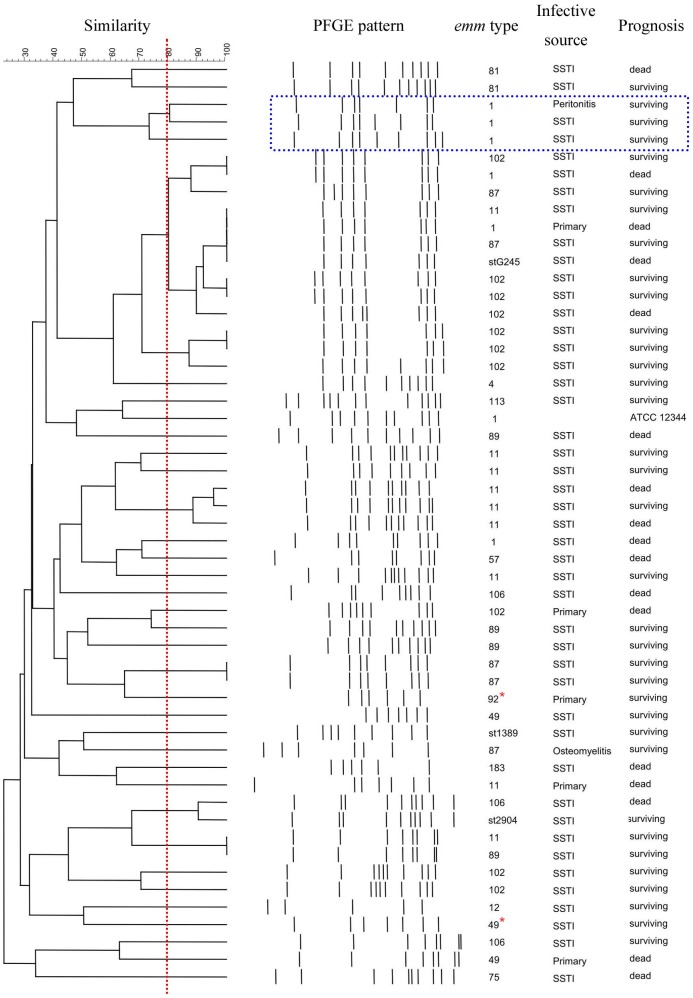
Dendrogram of pulsed-field gel electrophoresis (PFGE) among isolates of *Streptococcus pyogenes*. Thirty-five PFGE clusters with ≥80% similarity were identified among the 52 isolates of *S. pyogenes*. The majority of the isolates were grouped according to their *emm* type. Most *emm*102 belonged to the largest cluster. The nonfatal *emm*1 isolates were grouped together (in the rectangle outlined by a broken line) and distributed apart from the fatal isolates. *Streptococcus pyogenes* ATCC 12344 was used as a control strain. SSTI, skin and soft-tissue infection; *, isolates digested using Cfr9I.

Figure 3 shows the PFGE patterns and their relation to the *emm* types, infective sources, and prognoses of patients. Two isolates, comprising 1 *emm*49 and 1 *emm*92, were resistant to *Sma*I digestion, but they could be digested using the isoschizomer Cfr9I. However, 1 *emm*77 was still resistant to both *Sma*I and Cfr9I digestion. Among the 52 isolates, 35 PFGE clusters with ≥80% similarity were identified. The majority of the isolates were grouped according to their *emm* type. Most *emm*102 (70%) belonged to the largest cluster of PFGE. Half of *emm*1 isolates, which presented nonfatal infections, were grouped together, whereas the other fatal *emm*1 distributed away from the nonfatal cluster. There was no correlation between all the PFGE clusters and fatality (*P* = 0.324). No epidemiological correlation to the PFGE clusters could be established, such as residence, workplace, or time of presentation.

## Discussion

Here we report the results of an 8-year surveillance of STSS in a university hospital in southern Taiwan. This critical disease was not encountered frequently. Among 509,531 ED visits, only 53 patients were diagnosed with STSS. Recent population-based studies in Europe and the United States reported that STSS complicated approximately 10–20% of invasive GAS infections with an estimated incidence of 3.5 cases per 100,000 persons per year [19]–[23].

Most surveillance studies of invasive GAS infections have shown no significant difference in gender [19], [22], [23], [38]–[40], including those from Taiwan [41], [42]. However, a male predominance (81.1%) for STSS was observed in our study. An increased ratio of females among invasive GAS infections in young people was found in a report from Denmark [23], principally due to increased cases of puerperal sepsis in young females. In the present study, skin and soft-tissue infection was the major disease that resulted in STSS, and no puerperal sepsis was found in our patients. Our previous study describing GAS skin and soft-tissue infections also revealed a male predominance [28]. Therefore, the difference in the proportion of males to females may primarily be attributable to the nature of the original infection.

Patients aged 50–69 years have been reported to be prone to developing STSS in invasive GAS infections [20], [22]–[24]. Our study also revealed a similar result. Most of our patients had underlying medical diseases, such as diabetic mellitus, hypertension, gout, or liver cirrhosis. However, we identified 2 previously healthy young boys infected by GAS *emm*1 that died of STSS soon after presentation to our hospital [43]. In addition to microbiological characteristics, host features have been shown to impact the manifestation of GAS infections [44].

GAS *emm*1 has been recognized as the most important *emm* type to be associated with severe infections and fatal outcomes in the United States, Canada, Europe, and Japan [19]–[25]. Outbreaks of *emm*89 infections were also reported in some countries [19]–[21], [23], [25]. Our study demonstrated that *emm*102, *emm*11, *emm*1, *emm*87, and *emm*89 were the leading types which were associated with STSS. Type *emm*1 only contributed to 11.3% of STSS in our surveillance. The overall mortality rate of our patients was 32.1%, which was consistent with other studies [19]–[23]. Associations between mortality and some *emm* types have been recognized [19]–[23]. Although the results were not statistically significant, our study revealed a higher mortality rate of patients infected with *emm*1 and *emm*106, and a relatively lower fatality of *emm*102 infections. Moreover, the PFGE patterns of GAS distinguished the surviving and fatal clones among the *emm*1 and *emm*102 isolates. Further studies to understand the significance of these results are warranted.

The 26-valent M-based vaccine was developed on the basis of epidemiological data from North America [29]. Unfortunately, this vaccine is only effective against 41.5% of our isolates. The theoretical coverage of this vaccine against GAS in non-developed countries is low compared to high-income countries [5]. Recently, a new 30-valent vaccine has been constructed based on more extensive surveillance data of GAS infections from the USA, Europe, and some developing countries [30]. In experiments, the 30-valent vaccine evoked cross-opsonic bactericidal antibodies against the vaccine serotypes, as well as a significant number of non-vaccine serotypes of GAS [30], [31]. These results suggested the efficacy of the 30-valent vaccine could extend beyond the *emm*-types included in the vaccine. The main *emm* type in our surveillance, *emm*102, was not included in the 26- or 30-valent GAS vaccine, nor was it involved in the bactericidal test of the 30-valent vaccine [31]. As a result, whether *emm*102 is under the potential coverage of this new vaccine is not clear. However, *emm*102 was associated with a lower case-fatality rate than other major *emm* types in our study. It might be reasonable not to include *emm*102 in the GAS vaccine and leave room for more virulent *emm* types.

Different *emm* types can harbor particular SAg gene profiles [19], [35]. Several studies have described the association between the *speA* gene and severe GAS infection [19], [20], [24], [35]. However, the majority of *emm*1, which was the leading type that caused invasive GAS infections in many countries, was also found to frequently carry the *speA* gene [19], [20], [24], [35]. As a result, it would be difficult to discriminate between the roles of the *speA* gene and *emm*1 in the pathogenesis of invasive GAS infection. In our surveillance, most *emm*1 isolates also had the *speA* gene. However, no *speA* gene was found in isolates other than *emm*1 and *emm*11. Furthermore, rather than the *speA* gene, some studies have reported an association between invasive GAS infections and the *speC* gene [45], [46]. We could not draw conclusions about the role of SAgs and *emm* types in STSS in this observational study.

The proteins SpeB, SpeF, SpeG, SpeJ, and SmeZ were chromosomally encoded, whereas SpeA, SpeC, SpeH, SpeI, SpeK, SpeL, SpeM, and SSA were encoded on bacteriophages [9]. The chromosomally located genes are presumably identified frequently in GAS. However, *speF* was recognized at a low frequency in our study. Absence of this gene has been found in several studies, despite it's chromosomal origin [8], [23], [35]. Some authors questioned whether the absence of SAg genes might be false-negative due to inadequate primers that could not amplify the polymorphic alleles [12], [15], [47]. However, studies using the same methodology also revealed a diverse percentage on the detection of these genes [19], [48]. This exact reason is not clear; however, it may be due to a number of clones lacking these genes in some geographic areas [12], [15], [19], [23], [35].

Both *speG* and *speJ* are also considered to be chromosomal genes. Studies have revealed the absence of *speG* in some *emm* types [19], [23]. For example, only 30–50% of isolates carried *speG* in *emm*4 and *emm*77 from studies in Europe and the USA [19], [23]. *speG* was present in 81.9% of isolates in our study, which was similar to other studies [8], [12], [19], [23]. Some authors suggested that the contrasting results of *speG* carrying rate in studies were most likely due to different methodologies [12]. Regarding *speJ*, a low frequency of carrying rate was found in the majority of studies [8], [12], [15], [19], [23], [35], including our surveillance. Loss of *speJ* from the genome of GAS descendants was suggested after acquirement of this SAg, which was originally on a bacteriophage [49].

The genes *speH* and *speI* are located contiguously on the same bacteriophage (*Φ*370.2) [50], as well as *speL* and *speM* [(*ΦspeL/M*) [51]. Theoretically, the genes in adjacent positions on the same bacteriophage are expected to be detected together. However, *speH* and *speI* were observed independently in 12 isolates, and *speL* and *speM* were found independently in 4 isolates in our study. Several studies also revealed the genes in tandem on the same bacteriophage were not co-detected [12], [52], [53]. A possible reason for this condition is that these genes may be lost during integration into the genome [12], [52], [53].

There are limitations to this study, of which the small case number is the most important. Although this study was an 8-year surveillance, only 53 cases were included because STSS is still uncommon, despite the resurgence of invasive GAS infection. However, to the best of our knowledge, this surveillance is the largest report of STSS in Taiwan to date. Additionally, because the demographic information, laboratory findings, and underlying illnesses were collected retrospectively from medical records, these data may be incomplete, and some factors may not have been detected or explored fully for a statistical study.

In conclusion, despite the rarity of this illness, STSS constitutes a medical emergency with a high mortality rate. Our surveillance in southern Taiwan revealed an emergence of uncommon *emm* types, and these types, particularly *emm*102, were the leading types that caused STSS. We also revealed a different SAg gene distribution for these uncommon *emm* types. Characterization of the clinical, epidemiological, and molecular characteristics of STSS will help to improve our understanding of this life-threatening disease.
